# Reference Genes Selection and Normalization of Oxidative Stress Responsive Genes upon Different Temperature Stress Conditions in *Hypericum perforatum* L

**DOI:** 10.1371/journal.pone.0115206

**Published:** 2014-12-11

**Authors:** Isabel Velada, Carla Ragonezi, Birgit Arnholdt-Schmitt, Hélia Cardoso

**Affiliations:** EU Marie Curie Chair, ICAAM, Instituto de Ciências Agrárias e Ambientais Mediterrânicas, IIFA, Instituto de Investigação e Formação Avançada, Universidade de Évora, Ap. 94, 7006-554 Évora, Portugal; New Mexico State University, United States of America

## Abstract

Reverse transcription-quantitative real-time PCR (RT-qPCR) is a widely used technique for gene expression analysis. The reliability of this method depends largely on the suitable selection of stable reference genes for accurate data normalization. *Hypericum perforatum* L. (St. John's wort) is a field growing plant that is frequently exposed to a variety of adverse environmental stresses that can negatively affect its productivity. This widely known medicinal plant with broad pharmacological properties (anti-depressant, anti-tumor, anti-inflammatory, antiviral, antioxidant, anti-cancer, and antibacterial) has been overlooked with respect to the identification of reference genes suitable for RT-qPCR data normalization. In this study, 11 candidate reference genes were analyzed in *H. perforatum* plants subjected to cold and heat stresses. The expression stability of these genes was assessed using GeNorm, NormFinder and BestKeeper algorithms. The results revealed that the ranking of stability among the three algorithms showed only minor differences within each treatment. The best-ranked reference genes differed between cold- and heat-treated samples; nevertheless, *TUB* was the most stable gene in both experimental conditions. *GSA* and *GAPDH* were found to be reliable reference genes in cold-treated samples, while *GAPDH* showed low expression stability in heat-treated samples. *26SrRNA* and *H2A* had the highest stabilities in the heat assay, whereas *H2A* was less stable in the cold assay. Finally, *AOX1*, *AOX2*, *CAT1* and *CHS* genes, associated with plant stress responses and oxidative stress, were used as target genes to validate the reliability of identified reference genes. These target genes showed differential expression profiles over time in treated samples. This study not only is the first systematic analysis for the selection of suitable reference genes for RT-qPCR studies in *H. perforatum* subjected to temperature stress conditions, but may also provide valuable information about the roles of genes associated with temperature stress responses.

## Introduction

Gene expression analysis has been widely used as a method to study the complex signaling and metabolic pathways underlying cellular and developmental processes in biological organisms, including plants. Growing number of studies of expression levels of several genes in plants have been carried out in order to understand the cellular and molecular mechanisms involved in plant development and growth, as well as, in plant responses to biotic (pathogen infection) and abiotic (environmental) stresses [1]–[4].

The analysis of gene expression has been performed by using different methods such as, northern blotting, ribonuclease protection assay, reverse transcription-polymerase chain reaction (RT-PCR), reverse transcription-quantitative real-time PCR (RT-qPCR), DNA microarrays [5], and next generation sequencing (NGS) technologies [6]. These last three technologies in particular have gained a wider appeal for the quantification of gene expression. It is clear that microarrays and NGS are extremely popular due to the ability to perform high throughput analysis. It is also clear that because of their relative simplicity and portability, qPCR-based assays will continue to be in demand for some considerable time [6]. Moreover, NGS data is currently expanding in many plant species [7]–[9] and RT-qPCR provides a reliable method for validating such huge amount of RNA Sequencing (RNA-seq) data [10]. However, several variables need to be controlled to obtain reliable quantitative expression measures by RT-qPCR. These include variations in initial sample quantity, RNA recovery, RNA integrity, efficiency of cDNA synthesis, and differences in the overall transcriptional activity of the tissues or cells analyzed [11]. To overcome the problem of variability, a normalization step has to be used prior to gene expression analysis in order to minimize its effects. The most common approach to normalize RT-qPCR data is the introduction of reference genes (RG) [12]–[15]. A suitable reference gene is assumed to be unaffected by the experimental conditions and therefore should be expressed at a constant level among samples [16]. Consequently, any changes in its expression level are due only to technical variations which should be discounted from the variation of the target gene expression levels. Indeed, the purpose of a reference gene is to remove the technical variations, ending up with true biological changes [17]. The use of only one reference gene as well as the use of the most frequently used reference genes for normalization without a prior validation is no longer considered a good strategy [18]. It is widely recognized that the use of inappropriate RGs may result in misinterpretation of the expression pattern of a given target gene thereby introducing flaws in the understanding of the gene's role. Recently, efforts have been directed towards systematization and standardization of these type of analyses, and the MIQE (minimum information for publication of quantitative real-time PCR experiments) guidelines suggest the use of three reference genes to generate more reliable results [19]. The identification of stable RGs in various experimental designs will therefore contribute to have more accurate and reliable gene expression data.

Several algorithms such as GeNorm [20], NormFinder [11], BestKeeper [21], ΔCt [22], qBasePlus [23], as well as single-factor analysis of variance (ANOVA) and linear regression analysis [24] have been utilized. These algorithms have been applied in order to analyze the expression stability of candidate RGs among genotypes, organs, tissues, developmental stages and several biotic and abiotic stress conditions. Nowadays, a high diversity of plant species have been studied, including a number of important crops, vegetables and fruit plant species (Table 1).

**Table 1:** Previous studies on candidate reference genes selection for RT-qPCR data normalization in several crops, vegetables and fruit plants.

Plant species	Experimental condition (validated reference genes)	References
*Glycine max* L.	Seed development (*TUA5*, *UKN2*) and seed germination (Glyma05g37470 and Glyma08g28550).	[25]
	Different stresses (*EF1B* and *UKN2*).	[26]
*Zea mays* L.	Different tissues, developmental stages, and stress treatments (*CUL*, *FPGS*, *LUG*, *MEP* and *UBCP*).	[27]
	Respect to abiotic stresses, hormones across different tissue types (EF1a, b-TUB, and their combination - *EF1a+b-TUB*).	[28]
*Oryza sativa* L.	Different stress conditions (*EP* (LOC_Os05g08980), *HNR* (LOC_Os01g71770), and *TBC* (LOC_Os09g34040)).	[29]
*Solanum tuberosum* L.	Compatibility of potato–nematode interactions (*RPN7, UBP22, OXA1* and *MST2*).	[30]
	Tuber tissues exposed to cold treatments during different time periods (*ef1a* and *APRT*).	[31]
*S. lycopersicum* L.	Seed germination (SGN-U601022 (at2g20000), SGN-U580609 (at3g18780), SGN-U579915 (at4g02080), SGN-U569038 (at1g13320), SGN-U563892 (at5g25760), SGN-U568398 (at3g25800), SGN-U566667 (at5g46630), SGN-U567355, SGN-U584254 (at4g34270)).	[32]
*Brassica napus* L.	Different tissues and cultivars, and under different conditions. (*GDI1*, *PPR*, *UBA*, *OTP80* and *ENTH*).	[33]
*Lactuta sativa* L.	Different abiotic stresses (*MIR169*, *MIR171/170* and *MIR172*).	[34]
*Fragaria x ananassa*	Different tissues, cultivars, biotic stresses, ripening and senescent conditions, and SA/JA treatments (FaRIB413, *FaACTIN*, *FaEF1a* and *FaGAPDH2*).	[35]
*Citrullus lanatus*	Low temperature stress in leaves (*ClYLS8* and *ClPP2A*).	[36]
*Cucumis melo* L.	Leaves and roots under various stresses and growth regulator treatments (*CmRPL* and *CmADP*).	[37]
*Lycium* sp.	Fruit developmental stages (combination of *GAPDH* and *EF1a*).	[38]
	Ripening fruits (*EF1a* and *ACTIN1*) or plants under salt stress (*H2B1* and *H2B2*).	[39]
*Coffea* sp.	General assay (*GAPHD*, *Cycl*, and *UBQ10*), genotype (*GAPDH*, *UBQ10*, *Ap47*, and *EF*-*1A*), cold stress (*UBQ10*, *GAPDH*, *ACT*, and *EF*-*1A*), drought stress (*GAPDH*, *ACT*, *EF1A*, and *Apt*), multiple stress (*UBQ10*, *GAPDH*, *ACT*, and *elf-4A*).	[40]
	First hours of interaction (12, 48 and 72 hpi) with C. kahawae (*IDE*).	[41]
*Vitis vinifera* L.	First hours of interaction (0 h, 6, 12, 18 and 24 hpi) with P. viticola to study genotype and biotic stress effects (*UBQ*, *EF1a* and *GAPDH* (genotype effect), *EF1a*, *SAND* and *SMD3* (data normalization) and *EF1a*, *GAPDH* and *UBQ* Biotic stress effect).	[42]
*Citrus* sp.	Different citrus tissues (*18SrRNA*, *ACTB* and *rpII*).	[43]
*Musa acuminata*	Evaluation of robustness under different conditions, and in different tissues and varieties (*EF1*, *ACT*, and *TUB* (normalization in expression in leaves of greenhouse plants), *ACT* and *L2* (leaf discs), and combinations of *TUB*, *ACT*/*ACT11*, and *EF1* (expression studies in meristems)).	[44]
*Carica papaya* L.	Different experimental conditions (*EIF*, *TBP1* and *TBP2*).	[45]
*Pyrus pyrifolia* L.	Different environmental conditions, tissue types and developmental stages (*HIS*, *SAND*, *TIP).*	[10]
*Prunus persica* L.	Biotic stress treatments (*miR5059* and *miR5072*).	[46]
*Cocos nucifera* L.	Abiotic stress and endosperm developmental (*eEF1*-α and *UBC10*).	[47]

However, to the best of our knowledge, no studies were performed on the evaluation of the expression stability of candidate RGs in *Hypericum perforatum* L. (commonly named St. John's wort). Moreover, the few studies on gene expression by RT-qPCR performed in *H. perforatum* rely, even recently, on only one reference gene for data normalization [48]–[50]. Apart from these studies, no information is available on the most appropriate RGs to be used in data normalization in studies related with temperature stress response in this plant species.

*H. perforatum* is a widely known medicinal herb used mostly as a medication for depression [51] having also other broad pharmacological activities, such as anti-tumor, anti-inflammatory, antiviral, antioxidant, anti-cancer, and antibacterial properties [52], [53]. These properties are mainly due to the biosynthesis and accumulation of important secondary metabolites in its tissues. Naphtodianthrones, which include hypericin and pseudohypericins, and hyperforin, are the main biological active substances naturally present in *H. perforatum* [54]. *H. perforatum,* as a field growing plant, is frequently exposed to a variety of adverse environmental stresses which negatively affect both productivity and secondary metabolites content [55]. Environmental stress, such as extreme cold and heat stress (CS and HS, respectively), are two factors that greatly affect cultivated plants. The Mediterranean climate is characterized by high thermal amplitude, not only during a season, but also within a day, and therefore, plants need to suppress and respond quickly to the adverse effects of extreme temperature changes. It is important to understand how plants adapt to adverse temperatures in face of the current temperature changes that are occurring globally and that can critically affect plants behavior, such as physiological processes, crop production, and metabolite production.

Examples of genes which are associated with plant responses to abiotic stresses are, the alternative oxidase (*AOX1* and *AOX2*), the chalcone synthase (*CHS*) and catalase (*CAT*). AOX is a mitochondrial membrane protein that functions as terminal oxidase in the alternative (cyanide-resistant) respiratory pathway, where it reduces oxygen to water [56]. AOX relieves oxygen species (OS) originating from environmental stresses by limiting mitochondrial reactive oxygen species (ROS) formation and preventing specific components of the respiration chain from over-reduction [57] and canalizing ROS signals [58]. In dicot plant species AOX is nuclear encoded by a small multigene family composed by two sub-family genes, *AOX1* and *AOX2* [59]. CAT is a nuclear encoded enzyme which depending on its isoform could be localized in both the cytosol and peroxisomes [60] and probably also in the mitochondria [61]. CAT, the major H_2_O_2_-scavenging enzyme in all aerobic organisms [62], performs the rapid removal of H_2_O_2_ from the cell by oxidation of H_2_O_2_ to H_2_O and O_2_ [61]. CHS, a key enzyme of the flavonoid/isoflavonoid biosynthesis pathway and a component of the plant developmental program, is induced in plants under stress conditions such as UV light, bacterial or fungal infection [63].

For further development of RT-qPCR studies in *H. perforatum* that analyze the expression of stress responsive genes upon temperature stress conditions, the present study aimed to determine the most suitable RGs for data normalization. Here, we report a systematic analysis of eleven candidate RGs of *H. perforatum*, out of which some are commonly used as such, like the 18S ribosomal RNA (*18SrRNA*), the glyceraldehyde-3-phosphate dehydrogenase A subunit (*GAPDH*), and the beta-tubulin (*TUB*) [28], [33], but others not, such as the ribulose-1,5-bisphosphate carboxylase/oxygenase large subunit (*RBCL*), glutamate-1-semialdehyde 2,1-aminomutase (*GSA*), chamba phenolic oxidative coupling protein (*HYP1*), short-chain dehydrogenase/reductase (*SDR*), polyketide synthase 1 (*PKS1*) and polyketide synthase 2 (*PKS2*). The expression stabilities of these candidate RGs were evaluated using the three distinct statistical algorithms, GeNorm, NormFinder and BestKeeper, in order to determine the most stable and therefore most suitable genes for an accurate RT-qPCR normalization in *H. perforatum* upon temperature stress conditions.

## Materials and Methods

### Plant material and experimental conditions

*Hypericum perforatum* L. seeds were removed from the achenes of a mother plant growing under field conditions in the Alentejo region (Viana do Alentejo, Portugal, 38°21′37″N, 7°59′13″W). *H. perforatum* is not considered an endangered or protected species and no specific permissions were required for achene harvesting. After disinfection, seeds were *in vitro* inoculated (see details in [64]). Eight weeks after germination seedlings were transferred to 6.6×5.9 cm glass culture vessels (100 ml, Sigma-Aldrich, Sintra, Portugal) with fresh MS medium [65] (10 seedlings per flask). Cultures were maintained in a growth chamber at 16 h photoperiod, constant temperature of 25°C, and 80 µmol m^−2^s^−1^ of light intensity provided by day-light Philips fluorescent lamps. Nine-week-old seedlings were used for gene expression assays.

Two different experimental conditions were conducted in this study:

1) Cold stress (CS): cultures were transferred to 4°C. All the other parameters were maintained. Samples were collected at different time points: 0, 4, 8, 12, 24, 48 and 72 hours post incubation (hpi). Each biological sample consisted in a bulked sample of 10 seedlings. Three replicates were collected per time point.

2) Heat stress (HS): cultures were transferred to 35°C (the remaining parameters were maintained). Samples were collected at different time points: 0, 12, 24, 72 hpi and 7 days post incubation (dpi). Each biological sample consisted in a bulked sample of 3 seedlings. Three replicates were collected per time point.

### RNA isolation and first-strand cDNA synthesis

Total RNA was isolated with the RNeasy Plant Mini Kit (Qiagen, Hilden, Germany), according to the supplier's instructions and eluted in 30 µl volume of RNase-free water. To digest residual genomic DNA, RNA samples were treated on-column with DNase I (RNase-Free DNase Set, Qiagen, Hilden, Germany) following the manufacturer's instructions. The concentration of total RNA was determined with the NanoDrop-2000C spectrophotometer (Thermo Scientific, Wilmington, DE, USA) and its integrity analyzed by agarose gel electrophoresis (prepared with DEPC-treated water) after visualization of the two ribosomal subunits in a Gene Flash Bio Imaging system (Syngene, Cambridge, UK). A maximum of 1.5 µg of total RNA was used for reverse transcription with the Maxima First Strand cDNA Synthesis Kit for RT-qPCR (Thermo Scientific, Wilmington, DE, USA). All the subsequent procedures were performed according to the manufacturer's instructions.

### Quantitative real-time PCR

Relative quantification of gene expression was performed in the Applied Biosystems 7500 Real-Time PCR System (Applied Biosystems, Foster City, CA, USA). Real-time PCR reactions were carried out using 1× Maxima SYBR Green qPCR Master Mix, 300 nM of forward and reverse primers, and 1.25 ng of cDNA in a total volume of 18 µl. Primers for 11 candidate RGs and 4 target genes (Table 2) (designed from *H. perforatum* sequences deposited in National Center for Biotechnology Information - NCBI) were designed with the Primer Express v3.0 (Applied Biosystems, Foster City, CA, USA) using the default properties given by the software: amplicon length between 50 and 150 bp; primer length between 18 and 25 bp; melting temperature (Tm) of 60°C; guanine and cytosine (GC) content of 60%. All primer pairs were checked for their probability to form dimmers and secondary structures with the primer test tool of the software. cDNA samples were previously diluted in order to get the same concentration for all samples followed by a ten-fold dilution. The reactions were performed using the following thermal profile: 10 min at 95°C, and 40 cycles of 15 s at 95°C and 60 s at 60°C. No-template controls (NTCs) were used to assess contaminations and primer dimmers. A standard curve was performed using undiluted pool of all cDNA samples and three five-fold serial dilutions. All samples were run in duplicate. Melting curve analysis was done to ensure amplification of the specific amplicon. Quantification cycle (C_q_) values were acquired for each sample with the Applied Biosystems 7500 software (Applied Biosystems, Foster City, CA, USA).

**Table 2:** Gene ontology of candidate reference genes and target genes.

Gene	Accession Number	Complete name	Biological Process^1^	Cellular Component^1^	Molecular Function^1^
Candidate reference genes					
*Hp18SrRNA*	AF206934	*18S ribosomal RNA*	Translation**	Cytosolic small ribossomal subunit**	Structural constituent of ribosome**
*Hp26SrRNA*	DQ110887	*26S ribosomal RNA*	N/A	N/A	N/A
*HpGAPDH*	EU301783	*glyceraldehyde-3-phosphate dehydrogenase A subunit*	Calvin cycle*	Chloroplast, Membrane, Plastid*	oxidoreductase activity
*HpGSA*	KJ624985	*glutamate-1-semialdehyde 2,1-aminomutase*	Chlorophyll biosynthesis, Porphyrin biosynthesis*	Chloroplast, Plastid*	Isomerase*
*HpHYP1*	JF774163	*Chamba phenolic oxidative coupling protein*	Plant defense	N/A	Pathogenesis-related protein
*HpH2A*	EU034009	*histone 2A*	nucleosome assembly	nucleosome, nucleus	DNA binding
*HpPKS1*	EF186675	*polyketide synthase 1*	biosynthetic process	N/A	Transferase, Acyltransferase
*HpPKS2*	186676	*polyketide synthase 2*	biosynthetic process	N/A	Transferase, Acyltransferase
*HpRBCL*	HM850066	*ribulose-1,5-bisphosphate carboxylase/oxygenase large subunit*	reductive pentose-phosphate cycle, Photosynthesis, Carbon dioxide fixation, Calvin cycle	Plastid, Chloroplast	Oxidoreductase, Monooxygenase, Lyase
*HpSDR*	EU034010	*short-chain dehydrogenase/reductase*	Abscisic acid biosynthesis*	Cytoplasm*	Oxidoreductase*
*HpTUB*	KJ669725	*beta-tubulin*	microtubule-based process, protein polymerization*	Cytoplasm, Cytoskeleton, Microtubule*	GTPase activity, protein binding*
Target genes					
*HpAOX1*	EU330415	*Alternative oxidase 1*	Electron transport Respiratory chain Transport	Membrane	Oxidoreductase
*HpAOX2*	EU330413	*Alternative oxidase 2*	Electron transport Respiratory chain Transport	Membrane	Oxidoreductase
*HpCAT1*	AY173073	*Catalase-1*	hydrogen peroxide catabolic process	Cytoplasm*	Oxidoreductase Peroxidase
*HpCHS*	AF461105	*chalcone synthase*	biosynthetic process	endoplasmic reticulum, plant-type vacuole membrane, nucleus*	Transferase, Acyltransferase

1 From http://www.uniprot.org, N/A: not available. *Information obtained from the *Arabidopsis thaliana* NCBI protein accessions. **Information obtained from the *Arabidopsis thaliana* and provided by TAIR (www.arabidopsis.org).

### Determination of gene expression stability

To determine the expression stability for each of the candidate RGs, 3 different statistical algorithms were applied: GeNorm [20], NormFinder [11], and BestKeeper [21].

GeNorm determines the pairwise variation of every control gene with all other control genes as the standard deviation of the logarithmically transformed expression ratios, and defines the internal control gene-stability measure *M* as the average pairwise variation of a particular gene with all other control genes. GeNorm also determines the optimal number of genes required to calculate a reliable normalization factor [20]. The advantage of GeNorm is that it can find suitable combinations of reference genes required for normalization, which is more reliable than using only one reference gene. The main limitation of GeNorm is its sensitivity to co-regulation [66]–[68]. Co-regulated genes will have similar expression patterns having similar *M* values in the pairwise analysis of GeNorm, however, they might not have a stable expression in a specific tissue.

NormFinder enables estimation not only of the overall variation in the expression level of the candidate reference genes but also of the variation between sample subgroups of the sample set. It provides a direct measure for the estimated expression variation, enabling the user to evaluate the systematic error introduced when using the gene. The advantage of NormFinder is that it takes into account the inter- and intra-group variations and it shows less sensitivity to co-regulation of the candidate reference genes [11].

BestKeeper is an Excel based tool able to compare expression levels for up to ten reference genes together with ten target genes, each in up to hundred biological samples. It determines the ‘optimal’ reference genes employing the pair-wise correlation analysis of all pairs of candidate genes and calculates the geometric mean of the ‘best’ suited ones. The weighted index is correlated with up to ten target genes using the same pair-wise correlation analysis. While GeNorm software is restricted to the reference genes analysis only, in BestKeeper software, additionally up to ten target genes can be analyzed. Once a robust BestKeeper index is constructed, it can be applied as an expression standard in the same way like any single reference gene [21]. Both NormFinder and BestKeeper were used to avoid co-regulation and to compare the results obtained by these two software programs with those obtained by GeNorm, one of the most used software to determine suitable reference genes.

RefFinder is a user-friendly web-based comprehensive tool developed for evaluating and screening reference genes from extensive experimental datasets. It integrates the currently available major computational programs (GeNorm, NormFinder, BestKeeper, and the comparative ΔΔCt method) to compare and rank the tested candidate reference genes. Based on the rankings from each program, it assigns an appropriate weight to an individual gene and calculates the geometric mean of their weights for the overall final ranking (http://www.leonxie.com/referencegene.php). RefFinder was used here in order to obtain the expression stabilities values from GeNorm, NormFinder and BestKeeper in the same tool, instead of using each program separately.

The input data were the raw C_q_ values for each sample and each of the candidate RGs (these were organized in columns). For each candidate gene, the stability values from the GeNorm (or average expression stability value, *M*) and NormFinder algorithms were taken, as well as, the values of the standard deviation of C_q_ value and the Pearson coefficient of correlation (*r*) were taken from the BestKeeper algorithm. For each RG a weight was given which corresponds to the number of the position given by GeNorm, NormFinder and BestKeeper. That is, the most stable RG was assigned the number 1 and the least stable RG was assigned the number 11. By calculating the geometric mean of these values, a ranking of the RGs using the three algorithms together was obtained and designated in this work by geometric mean.

### Reference genes validation

In order to validate the candidate RGs, four target genes, namely *CHS, CAT1, AOX1* and *AOX2*, were selected from the literature based on their response to environmental stress conditions [63], [69]–[72]. For normalization of the target genes expression levels, C_q_ values were converted into relative quantities (RQ) by the delta-Ct method [20] using the formula RQ = E^ΔCq^, where E is the amplification efficiency calculated for each primer pair and ΔC_q_ = lowest C_q_ – sample C_q_. Amplification efficiency (E) was calculated using the formula E = 10^(-1/slope)^, where the slope was given by the Applied Biosystems (AB) software. A normalization factor was obtained by calculating the geometric mean between the relative quantities of the selected RGs (for each normalization strategy) for each sample. For each target gene, calculating the ratio between the relative quantities for each sample and the corresponding normalization factor, a normalized gene expression value was obtained. The graphs show the mean ± standard deviation of three biological replicates and correspond to the ratio between treated and untreated samples for each time point, with bars representing the fold-change related to control group (0 hpi), which was set to 1. Statistical significances (*p*≤0.05 and *p*≤0.01) between the two means were determined by the t-test using IBM SPSS Statistics version 22.0 (SPSS Inc., USA).

## Results

### Amplification specificity and efficiency

RNA samples were analyzed for their quantity and purity with a NanoDrop-2000C spectrophotometer (Thermo Scientific, Wilmington, DE, USA). All samples showed an absorbance ratio at 260/280 nm of above 1.8. Agarose gel electrophoresis revealed only the two rRNA subunits (18S and 28S) with well-defined bands and with no indication of RNA degradation (data not shown).

The amplification specificity of each gene was confirmed by performing a melting curve analysis after each PCR run. All the primer pairs used amplified the expected specific product and no formation of primer dimmers was observed (S1 Figure and S2 Figure). Amplification in NTCs was observed only for *18S* and *26S rRNAs* which was due to high transcript abundance and therefore confirmed by low C_q_ (C_q_<15) values (data not shown). PCR efficiency of each primer pair was calculated using a standard curve with four points: the undiluted pool, containing all cDNA samples, and three five-fold serial dilutions of the undiluted cDNA pool. The slope, y-intercepts, correlation coefficient (*r^2^*), and efficiency values were given by the AB software. The slope values ranged between −3.234 and −3.674 for CS assays, and between −3.259 and −3.574 for HS assays (data not shown). The correlation coefficient (*r^2^*) ranged from 0.991 to 0.999 for CS assays and from 0.993 to 1.00 for HS assays (except for *TUB* which has a lower value of 0.985) (Table 3). PCR efficiency, it ranged from 1.90 (90.50%) to 2.04 (103.81%) in CS assays (except for *PKS2* having only 87.15%), and from 1.90 (90.45%) to 2.03 (102.70%) in HS assays.

**Table 3:** Primers sequences for candidate reference genes and target genes and other parameters.

Gene (Accession Number)*	Gene	Primer Sequence (5′ – 3′)	Amplicon Length (bp)	Amplicon Tm (°C)	R^2^/E (%)**	
				Cold/Heat	Cold Stress	Heat Stress
Candidate reference genes						
AF206934	*Hp18SrRNA*	Fw: CGTCCCTGCCCTTTGTACAC Rv: CGAACACTTCACCGGACCAT	72	80.3/80.3	0.999/1.932 (93.18)	0.999/1.905 (90.46)
DQ110887	*Hp26SrRNA*	Fw: GCGTTCGAATTGTAGTCTGAAGAA Rv: CGGCACCCCCTTCCAA	65	80.8/81.0	0.998/1.966 (96.57)	0.999/1.924 (92.39)
EU301783	*HpGAPDH*	Fw: GGTCGACTTCAGGTGCAGTGA Rv: CACCATGTCGTCTCCCATCA	76	81.0/81.3	0.998/1.905 (90.49)	0.999/1.928 (92.78)
KJ624985	*HpGSA*	Fw: GCAATAATCCTTGAACCTGTTGTG Rv: CCTGCGGAGAGCGTTGA	78	78.4/78.7	0.998/2.011 (101.05)	0.997/1.982 (98.19)
JF774163	*HpHYP1*	Fw: GGAGGAAGCAAGGGTAAGATTACA Rv: CCCGATCTTGACTTCTTCTTCATT	81	77.0/77.0	0.998/1.967 (96.69)	0.999/1.968 (96.76)
EU034009	*HpH2A*	Fw: CCGGTTGGGAGGGTTCA Rv: TGCACCGACCCTTCCATT	63	79.7/79.7	0.997/1.988 (98.76)	0.998/1.967 (96.72)
EF186675	*HpPKS1*	Fw: ACGGACGCTGCCATCAA Rv: ACATAACCGTGTACCTTGTCTTCACA	76	79.2/78.9	0.991/2.018 (101.78)	0.996/1.958 (95.79)
186676	*HpPKS2*	Fw: GCCTGCGCGATTGTAGGA Rv: GCCCTTCACTAGCTCGAATATTG	66	80.7/81.5	0.991/1.871 (87.14)	0.997/1.965 (96.49)
HM850066	*HpRBCL*	Fw: CGCGGTGGGCTTGATTT Rv: CGATCCCTCCATCGCATAAA	71	76.9/76.9	0.999/1.935 (93.47)	1.000/1.953 (95.30)
EU034010	*HpSDR*	Fw: TCGACCAAGCCGACTTGTATC Rv: CACCAAATGCAATTCTTGAAAATATC	79	76.7/76.7	0.991/1.943 (94.32)	0.993/1.982 (98.23)
KJ669725	*HpTUB*	Fw: GGAGTACCCTGACAGAATGATGCT Rv: TTGTACGGCTCAACAACAGTATCC	80	78.0/78.1	0.999/1.991 (99.09)	0.985/1.919 (91.92)
Target genes						
EU330415	*HpAOX1*	Fw: TTGGACAATGGCAACATCGA Rv: GGGAGGTAGGCGCCAGTAGT	69	80.5/80.5	0.995/1.934 (93.41)	0.998/1.933 (93.33)
EU330413	*HpAOX2*	Fw: TCAACGCCTACTTTGTGATCTATCTC Rv: AATGGCCTCTTCTTCCAAATAGC	80	78.6/78.7	0.998/2.022 (102.17)	0.994/1.941 (94.13)
AY173073	*HpCAT1*	Fw: CGCTTCCTCAACAGATGGATTAG Rv: ACCCAGATGGCTCTGATTTCA	71	79.1/79.1	0.998/2.018 (101.82)	0.999/1.983 (98.27)
AF461105	*HpCHS*	Fw: GCGCTGCATCGATCATCA Rv: CAGCTCGAACAAGGGCTTTT	65	80.3/80.3	0.997/2.038 (103.81)	0.999/2.027 (102.70)
						

*NCBI accession number, R^2^: correlation coefficient, E: PCR efficiency.

**Figure 1:**
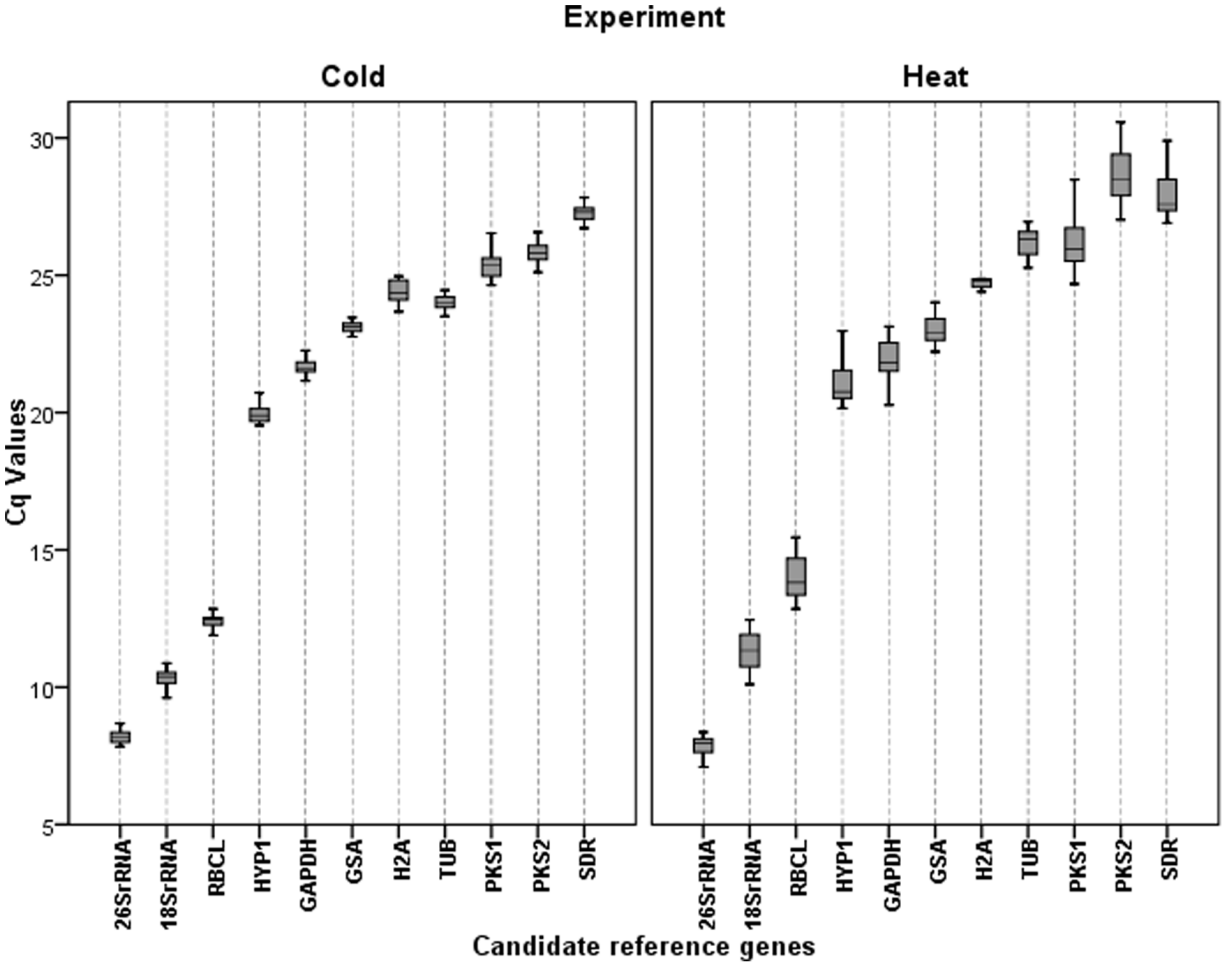
Range of C_q_ values of the candidate reference genes for each experimental condition. Each box corresponding to each candidate reference gene indicates the 25% and 75% percentiles. Whiskers represent the maximum and minimum values. The median is represented by the line across the box. *26SrRNA:* 26S ribosomal RNA*; 18SrRNA:* 18S ribosomal RNA*; RBCL:* ribulose-1,5-bisphosphate carboxylase/oxygenase large subunit*; HYP1:* Chamba phenolic oxidative coupling protein*; GAPDH:* glyceraldehyde-3-phosphate dehydrogenase A subunit*; GSA:* glutamate-1-semialdehyde 2,1-aminomutase*; H2A:* histone 2A*; TUB:* beta-tubulin*; PKS1:* polyketide synthase 1*; PKS2*: polyketide synthase 2*; SDR:* short-chain dehydrogenase/reductase.

The expression levels of the candidate RGs were determined as C_q_ values. The 11 tested genes showed a wide range of transcript abundance in both CS and HS assays (Fig. 1). The candidate genes showed a relatively narrow variation in their expression levels across all samples, particularly in CS assays, as shown in box-plots where the boxes and whiskers are smaller than in HS assays. *18SrRNA*, *26SrRNA* and *RBCL* were the most highly expressed genes for both CS and HS with the lowest mean C_q_ (mC_q_) values (mC_q_ between 7.97 and 13.91). *SDR* was the lowest expressed gene on both, CS and HS, showing the highest mC_q_ (27.30 for CS and 28.02 for HS). With the exception of the ribosomal genes and the *RBCL* presenting mC_q_ values around 8.0 and 14.0 for both assays, as mentioned above, the mC_q_ values ranged between approximately 20.0 and 28.0 for all other candidate, being the *HYP1* the most highly expressed gene with lower mC_q_ values for both CS and HS assays (19.98 and 21.29, respectively), and *SDR* the less expressed gene.

### Analysis of gene expression stability data

From the overall analysis, differences can be observed in the expression stability ranking of the candidate RGs between the two experimental conditions for each tested algorithm (Table 4). Additionally, differences can be also seen, although less significant, among the three algorithms applied within each assay. For CS experiments, GeNorm ranked *GAPDH* and *GSA* (M = 0.147) simultaneously as the most stable genes, and the *18SrRNA* (M = 0.166) as the third best gene. For NormFinder, the most stable gene was the *TUB* (SV = 0.104) followed by *18SrRNA* together with *26SrRNA* (SV = 0.121 for both genes) and *GAPDH* (SV = 0.124). The BestKeeper algorithm ranked *RBCL* (SD = 0.10, r = 0.213) first, followed by *TUB* (SD = 0.12, r = 0.641) and *26SrRNA* (SD = 0.12, r = 0.704) as the second and third ranked genes, respectively. From the geometric mean of all three algorithms, *TUB, GSA,* and *GAPDH*, were ranked in first, second and third positions, respectively.

**Table 4:** Candidate reference genes for cold and heat stresses determined by GeNorm, NormFinder, BesteKeeper, and by the combination of the 3 algorithms.

	COLD	COLD	COLD	COLD	HEAT	HEAT	HEAT	HEAT
Rank	GeNorm M	NormFinder SV	BestKeeper SD/*r*	Geometric Mean	GeNorm M	NormFinder SV	BestKeeper SD/r	Geometric Mean
1	*GAPDH | GSA* (0.147)	*TUB* (0.104)	*RBCL* (0.10*/*0.213)	*TUB* (2.154)	*26S | H2A* (0.174)	*TUB* (0.139)	*26S* (0.28/0.798)	*26S* (1.442)
2		*18S* (0.121)	*TUB* (0.12*/*0.641)	*GSA* (2.714)		*H2A* (0.207)	*H2A* (0.34/0.826)	*H2A* (1.587)
3	*18S* (0.166)	*26S* (0.121)	*26S* (0.12*/*0.704)	*GAPDH* (2.884)	*GSA* (0.336)	*26S* (0.262)	*18S* (0.35*/*0.494)	*TUB* (2.884)
4	*26S* (0.179)	*GAPDH* (0.124)	*GSA* (0.16/0.697)	*26S* (3.302)	*SDR* (0.425)	*RBCL* (0.338)	*TUB* (0.42*/*0.912)	*18S* (4.932)
5	*TUB* (0.189)	*GSA* (0.152)	*HYP1* (0.17*/*0.001)	*18S* (3.476)	*HYP1* (0.478)	*18S* (0.473)	*RBCL* (0.54*/*0.760)	*GSA* (5.013)
6	*SDR* (0.199)	*SDR* (0.156)	*GAPDH* (0.17/0.705)	*RBCL* (3.826)	*TUB* (0.528)	*GSA* (0.561)	*PKS1* (0.56*/*0.638)	*RBCL* (5.192)
7	*H2A* (0.208)	*RBCL* (0.169)	*18S* (0.21*/*0.925)	*SDR* (6.604)	*RBCL* (0.584)	*PKS1* (0.595)	*GSA* (0.57*/*0.634)	*SDR* (7.368)
8	*RBCL* (0.219)	*H2A* (0.240)	*SDR* (0.23*/*0.925)	*HYP1* (7.399)	*18S* (0.617)	*GAPDH* (0.648)	*GAPDH* (0.67*/*0.805)	*HYP1* (7.399)
9	*HYP1* (0.250)	*HYP1* (0.339)	*H2A* (0.26*/*0.897)	*H2A* (7.958)	*GAPDH* (0.650)	*HYP1* (0.716)	*HYP1* (0.75*/*0.814)	*PKS1* (7.489)
10	*PKS2* (0.283)	*PKS2* (0.384)	*PKS2* (0.29*/*0.407)	*PKS2* (10.00)	*PKS1* (0.686)	*SDR* (0.764)	*SDR* (0.76*/*0.666)	*GAPDH* (8.320)
11	*PKS1* (0.328)	*PKS1* (0.486)	*PKS1* (0.30*/*0.365)	*PKS1* (11.00)	*PKS2* (0.828)	*PKS2* (1.416)	*PKS2* (0.87*/*0.001)	*PKS2* (11.00)

*M*: expression stability average; SV: stability value; SD: standard deviation of C_q_ value; *r*: Pearson coefficient of correlation.

For HS tolerance assays, *26SrRNA* and *H2A* were ranked in the two first positions with GeNorm (M = 0.174 for both genes) and BestKeeper (SD = 0.28, r = 0.798, SD = 0.34, r = 0.826, respectively). NormFinder ranked *TUB* in the first position (SV = 0.139) and *H2A* and *26SrRNA* were selected as the second and the third best genes, respectively (SV = 0.207 and SV = 0.262, respectively). The third ranked RG by GeNorm was the *GSA* (M = 0.336) and by BestKeeper was the *18SrRNA* (SD = 0.35, r = 0.494). For the overall final ranking, obtained by calculating the geometric mean of the ranked genes by the three algorithms, the three top RGs for the HS assay were the *26SrRNA*, *H2A*, and *TUB*.

### Determination of the optimal number of reference genes for normalization by GeNorm

**Figure 2:**
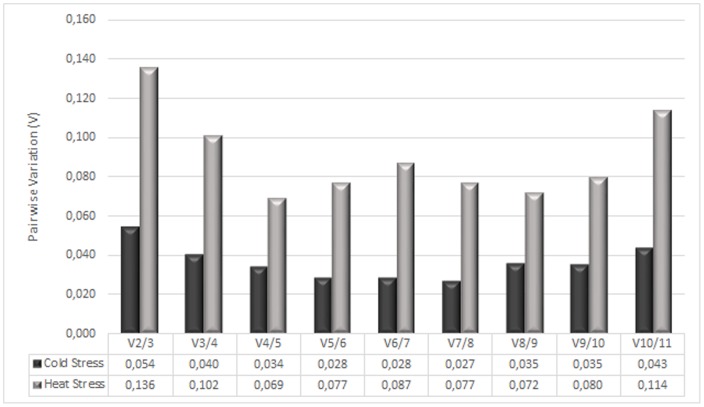
Determination of the optimal number of reference genes for normalization by pairwise variation using GeNorm.

The Genorm pairwise variation (V) values were determined for the candidate RGs in both experimental designs (CS and HS) using a cut-off value of 0.15, below which the inclusion of an additional RG is not required for normalization (Fig. 2). Interestingly, the optimal number of RGs used for normalization was only two for both CS assay (V2/3 = 0.054) and HS assay (V2/3 = 0.136), being both V values below the 0.15 cut-off value.

### Expression analysis of target genes for reference genes validation

**Figure 3:**
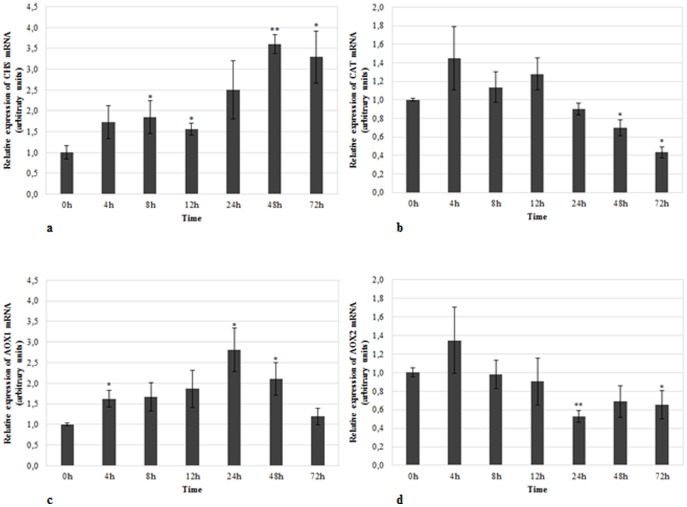
Relative mRNA expression of target genes in cold-treated samples. Expression of **a**) *CHS*, **b**) *CAT*, **c**) *AOX1*, and **d**) *AOX2* in cold-treated samples using *TUB*, *GSA* and *GAPDH* as reference genes in data normalization. The relative expression values are depicted as the mean ± standard deviation of three biological replicates and correspond to the ratio between treated and untreated samples for each time point. The bars represent the fold-change related to control group (0 hours) which was set to 1. Statistical significances (*p≤0.05 and **p≤0.01) between the two means were determined by the t-test using IBM SPSS Statistics version 22.0 (SPSS Inc., USA).

In order to validate the suitability of the selected candidate RGs, the expression profile of 4 target genes was analyzed in the present work. The target genes, selected from the literature based on their reaction to temperature stress response and oxidative stress [63], [69]–[72], were *CHS*, *CAT1*, *AOX1* and *AOX2*. A single normalization strategy was applied to both assays, based on the geometric mean of the three algorithms by using the 3 top-ranked RGs. *TUB*, *GSA* and *GAPDH* were used as RGs for data normalization in CS whilst *TUB* together with *26S* and *H2A* were used to normalize the expression results in HS assays. In CS assays, an accumulation of the *CHS* transcript was observed until 48 hpi with a 3.6 fold-change (*p*≤0.01) (Fig. 3a). From 24 hpi to 72 hpi, a down-regulation in the *CAT1* mRNA expression for roughly half (*p*≤0.05) of the corresponding expression in the control group was observed (Fig. 3b). *AOX1* transcript expression showed a gradual up-regulation in cold-treated samples until 24 hpi of about 2.8-fold (*p*≤0.05) with a following expression recovery until 72 hpi (Fig. 3c). *AOX2* mRNA showed a reduction in its expression after 24 hpi in 1.9-fold (*p*≤0.01), with a further slight tendency to recover (Fig. 3d).

**Figure 4:**
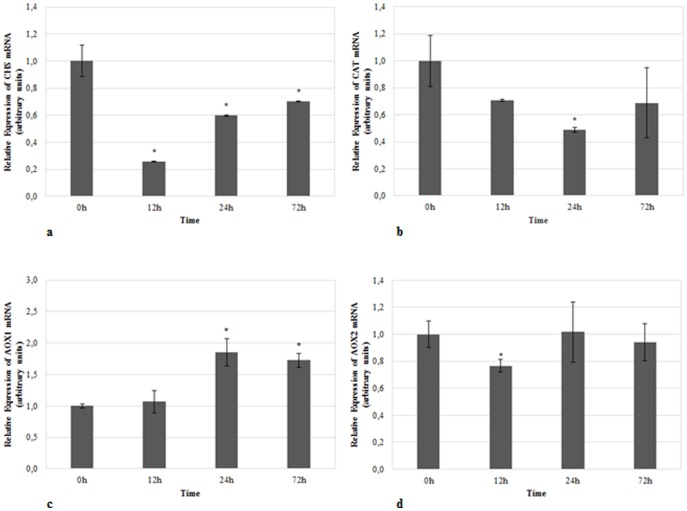
Relative mRNA expression of target genes in heat-treated samples. Expression of **a**) *CHS*, **b**) *CAT*, **c**) *AOX1*, and **d**) *AOX2* in heat-treated samples using *TUB*, *26S* and *H2A* as reference genes in data normalization. The relative expression values are depicted as the mean ± standard deviation of three biological replicates and correspond to the ratio between treated and untreated samples for each time point. The bars represent the fold-change related to control group (0 hours) which was set to 1. Statistical significances (*p≤0.05 and **p≤0.01) between the two means were determined by the t-test using IBM SPSS Statistics version 22.0 (SPSS Inc., USA).

In HS assays, our results showed a down-regulation in the *CHS* at 12 hpi of 3.8 fold-change (*p*≤0.01) with a subsequent gradual recovery until 72 hpi (Fig. 4a). Similarly in CS, the mRNA expression of *CAT1* had a tendency to decrease in heat-treated samples and after 24 hpi the value was around half (*p*≤0.05) of the expression in the control group (Fig. 4b). For *AOX1*, a slight increase in the mRNA expression of 1.8 fold-change (*p*≤0.05) was observed after 24 hpi, and this expression was maintained until 72 hpi (Fig. 4c). Although statistically significant (*p*≤0.05), the reduction in the *AOX2* mRNA expression after 12 hpi was very modest demonstrating, in general, a high stability of this gene over time (Fig. 4d).

## Discussion

In *H. perforatum*, studies on the expression stability of candidate RGs in a variety of experimental contexts has received no attention, and even recently only one RG is being used in RT-qPCR data normalization in this plant species [48]–[50] neglecting the knowledge that such a strategy is no longer admissible [19], [20]. Despite the pharmacological interest of the compounds produced by *H. perforatum*, very limited genomic information is available in NCBI. This is reflected by the absence of sequence data on a large number of frequently used genes. Consequently, for this study, a significant number of the genes selected had been recently isolated by members of our research group (*GAPDH*: EU301783, *GSA*: KJ624985, *H2A*: EU034009, *SDR*: EU034010, *TUB*: KJ669725, *AOX1*: EU330415, *AOX2*: EU330413). This study is the first detailed evaluation of the expression stability of several candidate RGs to be used for normalization in RT-qPCR studies in *H. perforatum* upon different stressful temperature conditions. Our analysis was based on the three most frequently used mathematical softwares, GeNorm, NormFinder and BestKeeper. Instead of using each software separately, the RefFinder tool was used here, since it integrates all three algorithms. We tested the expression stability of 11 candidate RGs, including commonly used RGs (*TUB, GAPDH, and 18SrRNA*), as well as, less frequently used RGs (*HYP1, GSA, H2A, PKS1, PKS2, RBCL, SDR, and 26SrRNA*). Within each algorithm, differences in the expression stability of the candidate genes were found when comparing the two experimental conditions of our study. These results reinforce previous conclusions, achieved by several authors using different plant species and experimental conditions, stating that the selection of the most stable RGs is highly dependent on the plant species and the experimental context [37], [73]–[75] and, therefore, assuming the existence of general RGs which can be used in different experimental situations could be of great risk. For example *GAPDH*, a commonly used RG, is ranked in first position by GeNorm in CS experiments, together with *GSA*. However, in heat treatment, this gene appeared to be an unsuitable RG ranking only in ninth position by the same algorithm. *GAPDH* has been also reported by others as a suitable RG, for example in *Chlamydomonas* during freezing acclimation [76] and in coffee upon CS [40]. However, it is not recommended for normalization of gene expression data in several studies involving temperature stress response and acclimatization performed in other plant species [29], [45], [77]. Another example from our study is the *H2A*, which is in final rankings in CS. However, in contrast, this gene is identified as one of the most stable RGs in HS, using the three algorithms.

In addition to the differences encountered in the selection of the most stable genes between the two experimental conditions, we also found some slight differences amongst the distinct algorithms tested. For example, *RBCL* was ranked in the first position by BestKeeper and therefore considered a good candidate in CS. However, it showed high expression variability using both NormFinder and GeNorm, staying only in seventh and eighth positions, respectively. One example, in HS, is the case of *SDR*, which is ranked in fourth position by GeNorm, but ranked only in tenth position by both NormFinder and BestKeeper. Similarly, results from other authors also demonstrate the existence of minor differences among distinct algorithms [13], [33], [38], and that are most probably due to the differences in the statistical algorithms. Nevertheless, taking into account the existence of some differences between algorithms, and additionally to overcome different limitations of each algorithm studied here, the stability of candidate RGs was also determined based on the geometric mean of all three statistical algorithms. Indeed, depending on the software used, the ranking of candidate reference genes can be slightly different [40], [78], [79]. Although many statistical approaches exist to determine both the stability of gene expression and to select the most appropriate RGs, to date, there is no consensus on which approach gives valid results. Thus, some authors consider the best approach is to combine the distinct algorithms for the selection of the most reliable reference genes in order to reduce the risk of artificial selection [80]. In fact, the simultaneous use of more than one algorithm has been also noted by other authors [67], [81] as producing highly correlated results and thus represents a good strategy for the selection of RGs for RT-qPCR normalization. In this study, the normalization strategy adopted to analyze the expression levels of the target genes to validate the RGs was based on the geometric mean of all three algorithms. Indeed, the software programs such as GeNorm, NormFinder and BestKeeper exist to determine the stability of the candidate RGs. However, the suitability of these genes should be evaluated with target genes associated with the experimental conditions in order to obtain reliable results [34]. The target genes used here were the *CHS*, *CAT1*, *AOX1* and *AOX2*, which encode for proteins, documented in the literature, as associated with plant stress responses and oxidative stress. From our study, *AOX1* transcripts were revealed to be affected by both cold and heat stressful temperatures, presenting an up-regulation in both conditions. In recent years, transgenic plants overexpressing *AOX1* genes have provided molecular evidence that *AOX* improves cold tolerance [69], [71]. *AOX* was proposed, in a hypothesis-driven approach, as target to develop functional markers for general stress tolerance across species and stresses [82], [83]. Extensive reports have shown that the expression of the *AOX1* gene is highly responsive to environmental stress, such as CS and HS, whilst *AOX2* members are generally not responsive, or are much less responsive (see reviews [84], [85]). Indeed, from our results, *AOX2* showed high expression stability in heat-treated samples. However, in contrast, this gene showed differential expression, being down-regulated in cold-treated samples. Both AOX and CAT are two antioxidative enzymes already described as being highly involved in ROS detoxification [86]. Our results demonstrate that despite a slight tendency for *CAT1* transcript to be up-regulated (not statistically significant) in CS for the first time points, *CAT1* mRNA is down-regulated both in CS and HS. Previous reports on the effects of stress on CAT activities vary depending on experiment and plant species or cultivars. Decreases in CAT activity have been reported upon HS in various plant species [87]–[90], in contrast with other studies which reported increases in CAT activity [91]–[98]. An increase in the CAT activity has also been reported upon CS [72], [99]–[103]. Nevertheless, the majority of studies available have focused on protein content and activity, with very few studies analyzing the link between transcript accumulation and protein. Locato et al. [97] have reported that in *Nicotiana tabacum* there is a decrease in transcript accumulation upon HS starting at 4 h after HS exposition. These authors suggest that under those experimental conditions, the regulation of *CAT* occurs with a mechanism acting downstream gene expression. In the same way, Watanabe and co-workers [104] revealed that in *Arabidopsis thaliana* a decrease in *CAT1* transcript level was detected 20 h after CS exposure. Mhamdi and co-workers [105] suggested that a transcriptional down-regulation of *CAT* could be important to induce or sustain increased H_2_O_2_ availability necessary for certain environmental responses or developmental processes. Our data are in agreement with these previous results, suggesting that the molecular mechanisms associated with temperature stress response involving differential expression of *CAT* mRNA encountered both in *N. tabacum* and in *A. thaliana* might be also found in *H. perforatum*. In contrast, previous studies from Skyba et al. [49] demonstrated, in *H. perforatum*, an increase in *CAT* transcript accumulation upon a low-temperature associated stressor, being the results genotype-dependent. The similar expression profile of *CAT1* and *AOX2* in CS, shown by our study, in which both are down-regulated, supports current knowledge of these proteins regarding their common involvement in ROS detoxification [86]. Worth a notice, our results suggest an earlier involvement of *AOX2* than *CAT1* in this process, as shown by the *AOX2* having the lowest mRNA expression at a time point earlier than *CAT1*.

We observed an increase in the *CHS* transcript accumulation upon CS. It is known that *CHS* can be induced in response to a diversity of biotic and abiotic stress factors, thus resulting in enhanced production of secondary metabolites (see review in [63]). Interestingly, we found a similar expression profile for *CHS* and *AOX1* in CS, and even for HS, with the exception of the first time point (12 hpi) for which a marked reduction was observed for *CHS*, from this time point on, both genes are similarly up-regulated. These findings corroborate the results obtained by Fiorani and co-workers [106] who reported a relationship between *AOX1a and CHS*, demonstrating that, at low temperature, transgenic *A. thaliana* overexpressing *AOX1a* shows enhanced transcription of *CHS*, a key gene of the polyketide phenylpropanoids biosynthesis [63].

In summary, to the best of our know ledge, this is the first study in which a set of candidate reference genes are analyzed in terms of their expression stability, by three distinct mathematical algorithms, in order to be used in RT-qPCR data normalization with *H. perforatum* submitted to stressful temperature conditions. Our results demonstrate considerable differences in the gene expression stability between cold and heat stress treatments, highlighting the importance of performing a selection of the most suitable reference genes prior to the RT-qPCR studies for each experimental condition. Our data also show that the reference gene ranking obtained by GeNorm, NormFinder and BestKeeper were overall very similar and presenting only slight differences. The present study pointed to *TUB* as a suitable RG in RT-qPCR data normalization in temperature-treated *H. perforatum* plants. Additionally, *GSA* and *GAPDH* are recommended for data normalization in studies of cold treatment, whilst *26SrRNA* and *H2A* are suggested for studies of heat treatment. The expression of the target genes *CHS*, *CAT1*, *AOX1* and *AOX2* were analyzed in order to emphasize the importance of validating the reference genes. They showed differential expression in both CS/HS experimental conditions, showing consistent results with what is known about the involvement of these genes in plant response to stressful temperatures, allowing the validation of the identified candidate reference genes.

## Supporting Information

S1 Figure **Melting curves of the 11 candidate reference genes and 4 target genes tested in cold assays.**
(PDF)

S2 Figure **Melting curves of the 11 candidate reference genes and 4 target genes tested in heat assays.**
(PDF)
