# Correction: Reference Genes Selection and Normalization of Oxidative Stress Responsive Genes upon Different Temperature Stress Conditions in *Hypericum perforatum* L

**DOI:** 10.1371/journal.pone.0119982

**Published:** 2015-03-26

**Authors:** The PLOS ONE Staff

There is an error in Fig. 3, “Relative mRNA expression of target genes in cold-treated samples,” Panel B, as well as in the legend for Fig 3. Please see the corrected Fig. 3 and its legend here.

**Fig 3:**
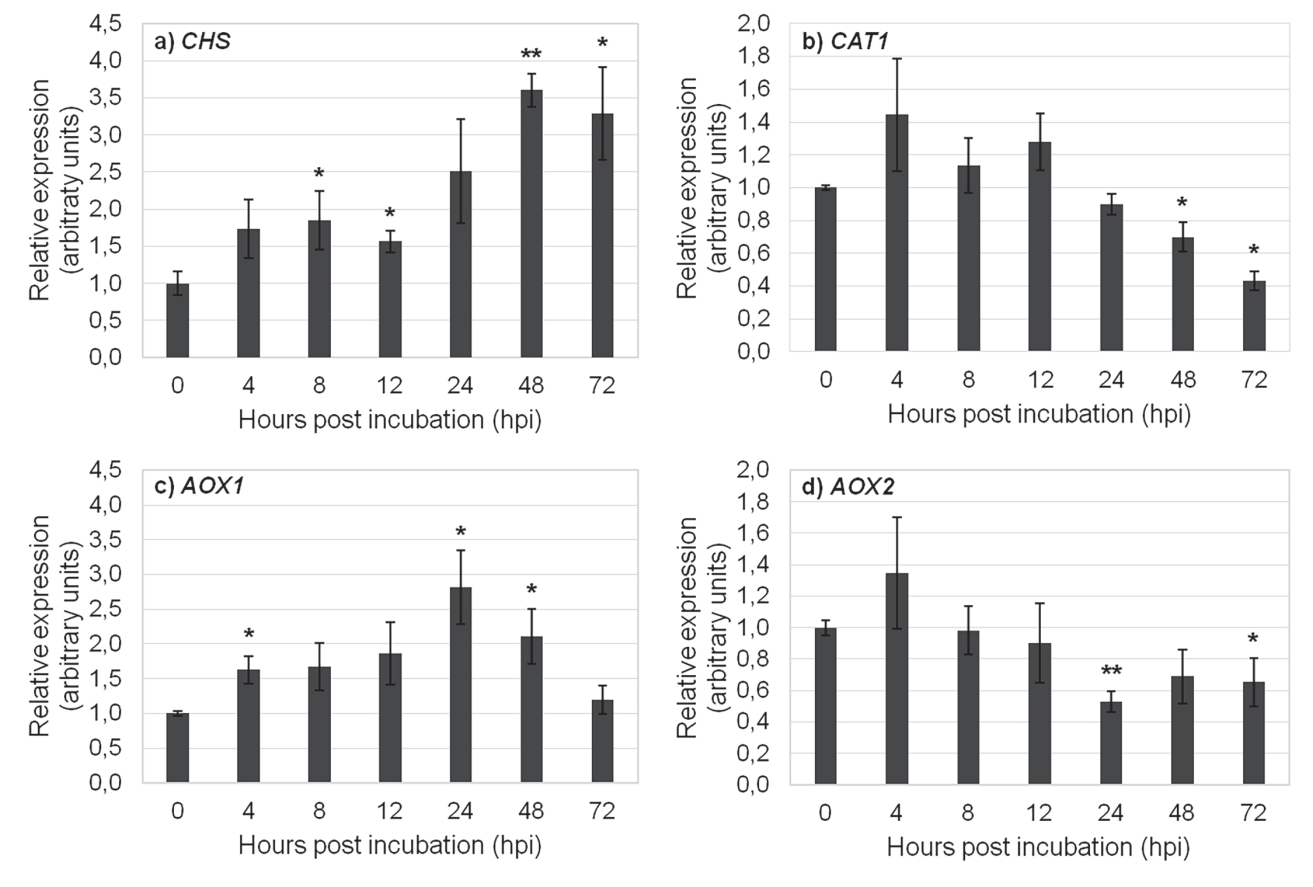
Relative mRNA expression of target genes in cold-treated samples. Expression of a) *CHS*, b) *CAT1*, c) *AOX1*, and d) *AOX2* in cold-treated samples using *TUB*, *GSA* and *GAPDH* as reference genes in data normalization. The relative expression values are depicted as the mean ± standard deviation of three biological replicates and correspond to the ratio between treated and untreated samples for each time point. The bars represent the fold-change related to control group (0 hours) which was set to 1. Statistical significances (*p≤0.05 and **p≤0.01) between the two means were determined by the t-test using IBM SPSS Statistics version 22.0 (SPSS Inc., USA).

There is an error in Fig. 4, “Relative mRNA expression of target genes in heat-treated samples,” Panel B, as well as in the legend for Fig. 4. Please see the corrected Fig. 4 and its legend here.

**Fig 4:**
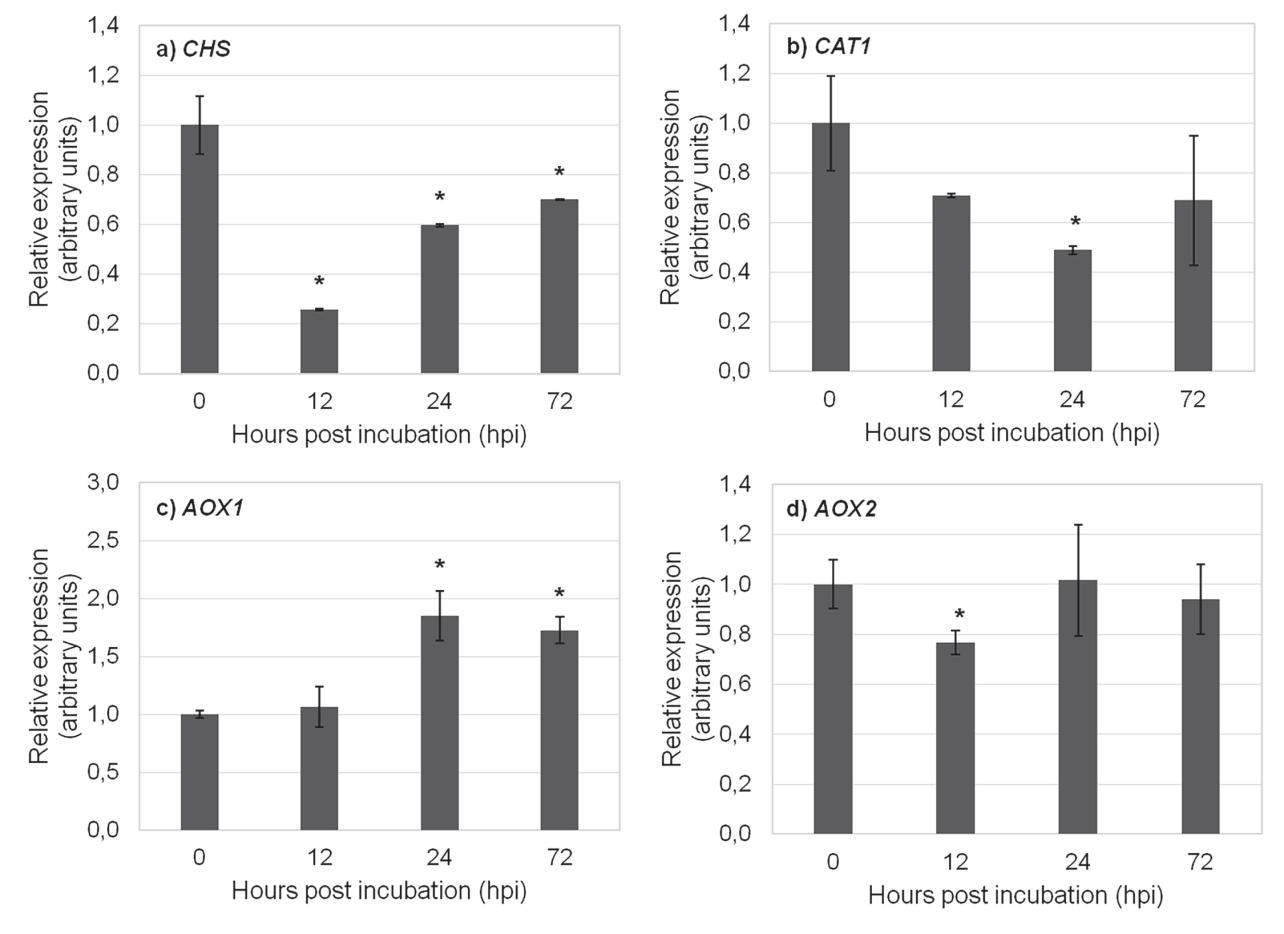
Relative mRNA expression of target genes in heat-treated samples. Expression of a) *CHS*, b) *CAT1*, c) *AOX1*, and d) *AOX2* in heat-treated samples using *TUB*, *26S* and *H2A* as reference genes in data normalization. The relative expression values are depicted as the mean ± standard deviation of three biological replicates and correspond to the ratio between treated and untreated samples for each time point. The bars represent the fold-change related to control group (0 hours) which was set to 1. Statistical significances (*p≤0.05 and **p≤0.01) between the two means were determined by the t-test using IBM SPSS Statistics version 22.0 (SPSS Inc., USA).
