# Supplementary figures and images for: Reference Genes Selection and Normalization of Oxidative Stress Responsive Genes upon Different Temperature Stress Conditions in Hypericum perforatum L
Source: PLoS One. 2014 Dec 11;9(12):e115206. doi: 10.1371/journal.pone.0115206 (PMC4263753; doi:10.1371/journal.pone.0115206)

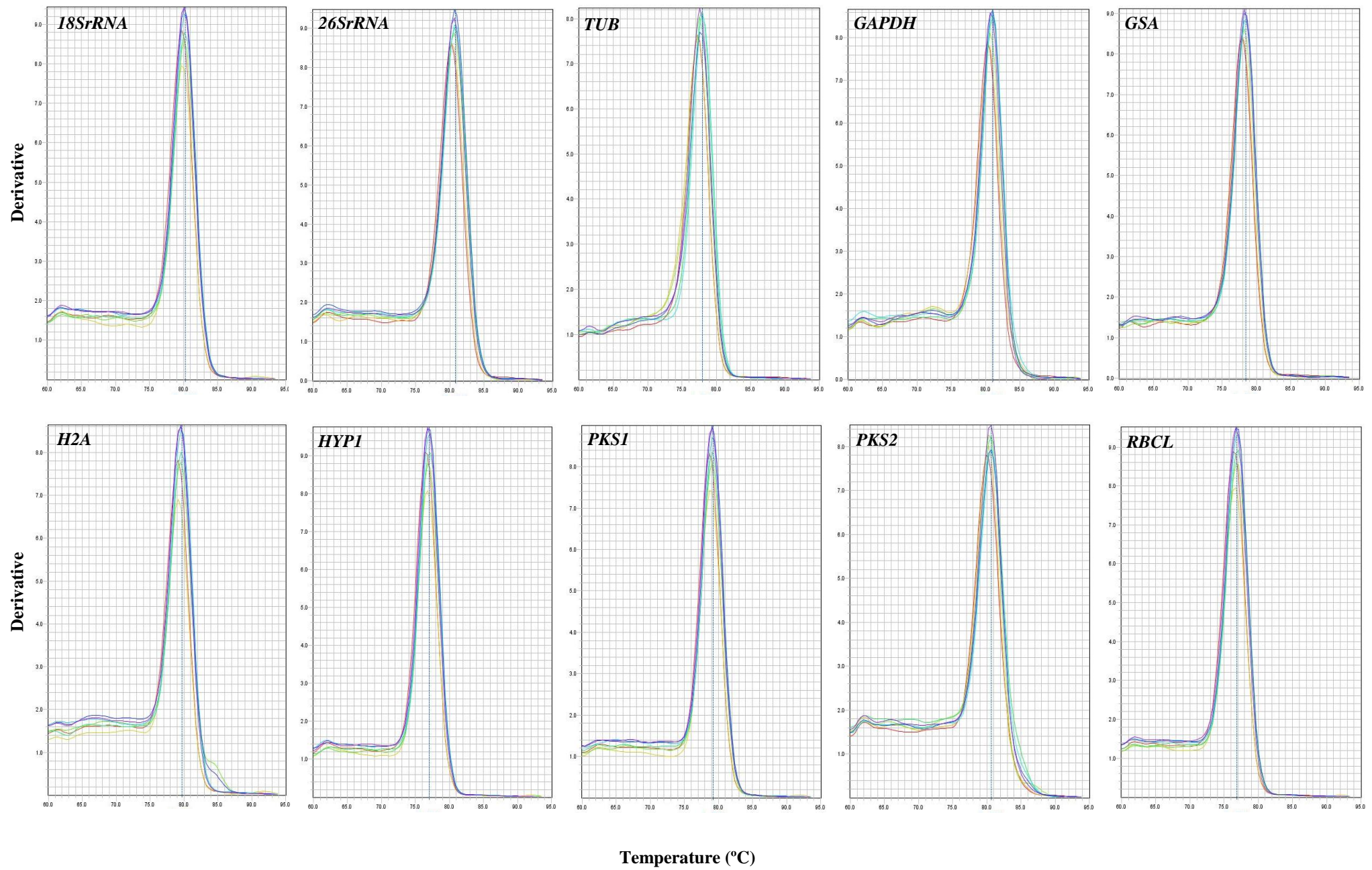

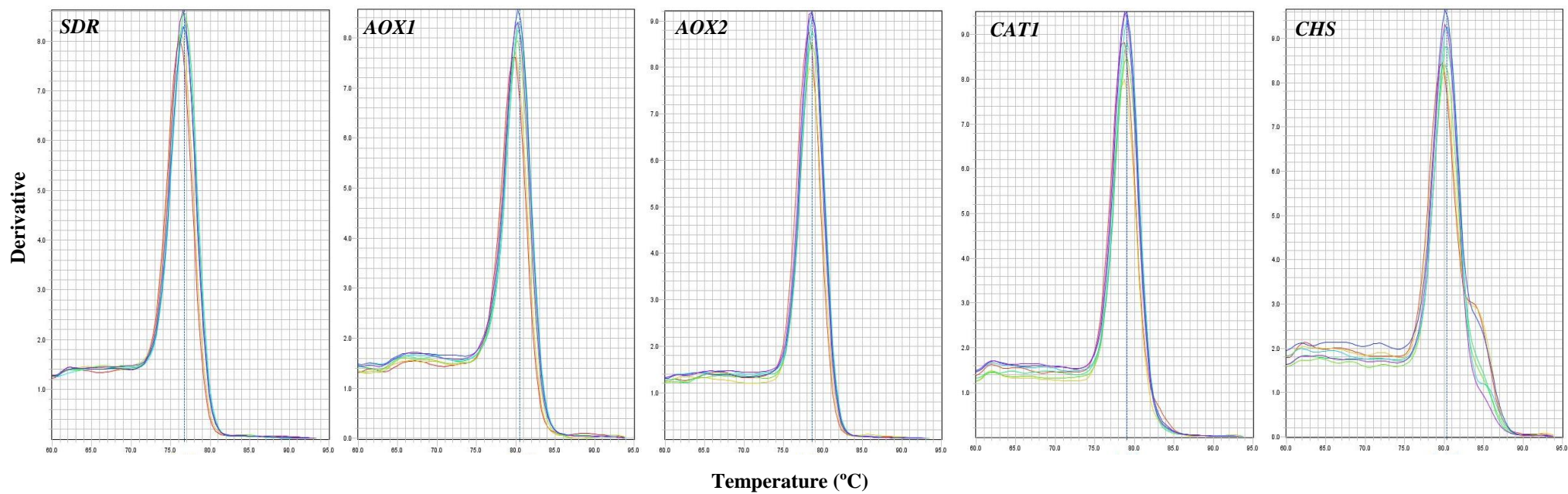

Supplement: S1 Figure — Melting curves of the 11 candidate reference genes and 4 target genes tested in cold assays. (PDF) [file pone.0115206.s001.pdf]

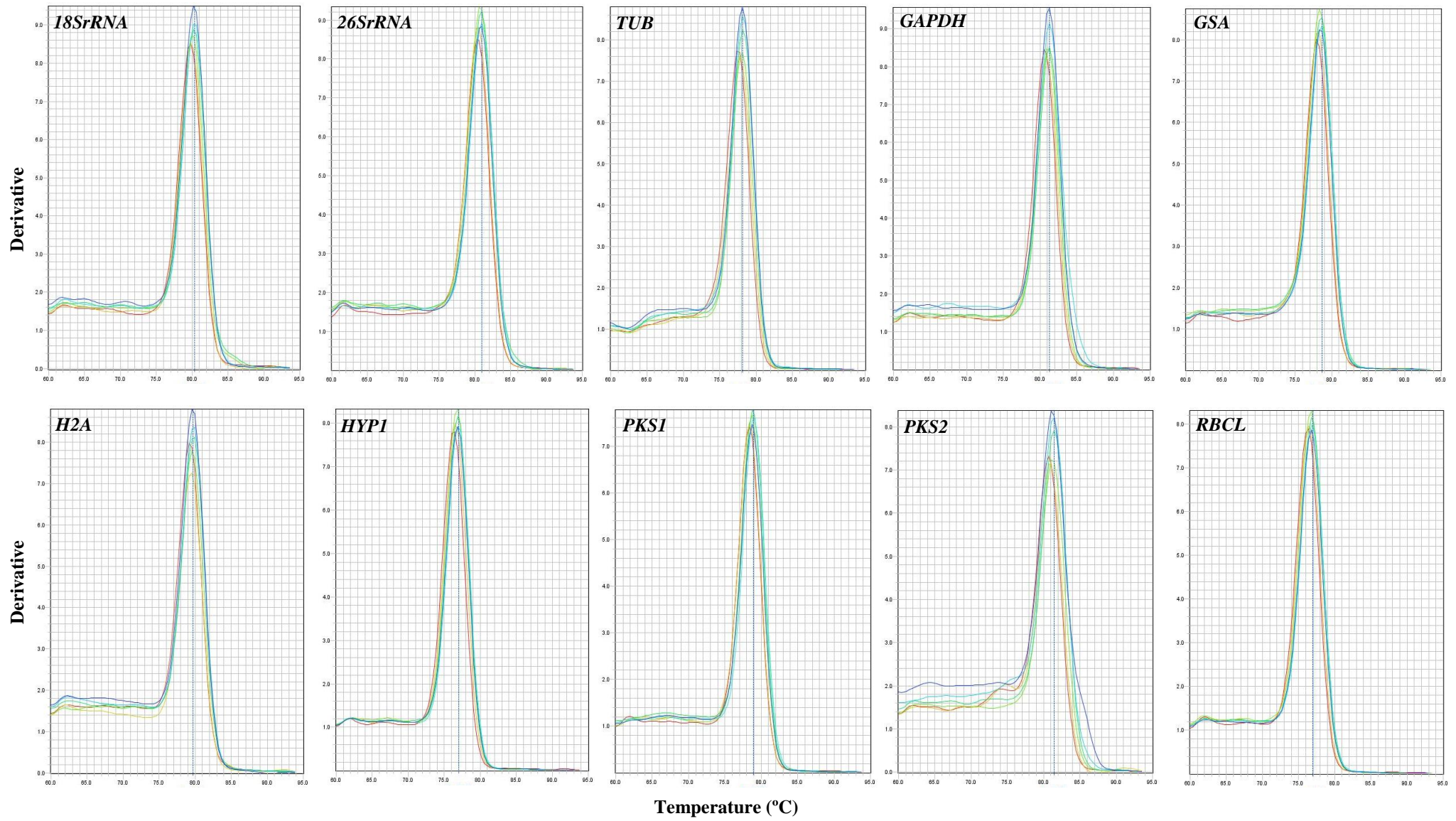

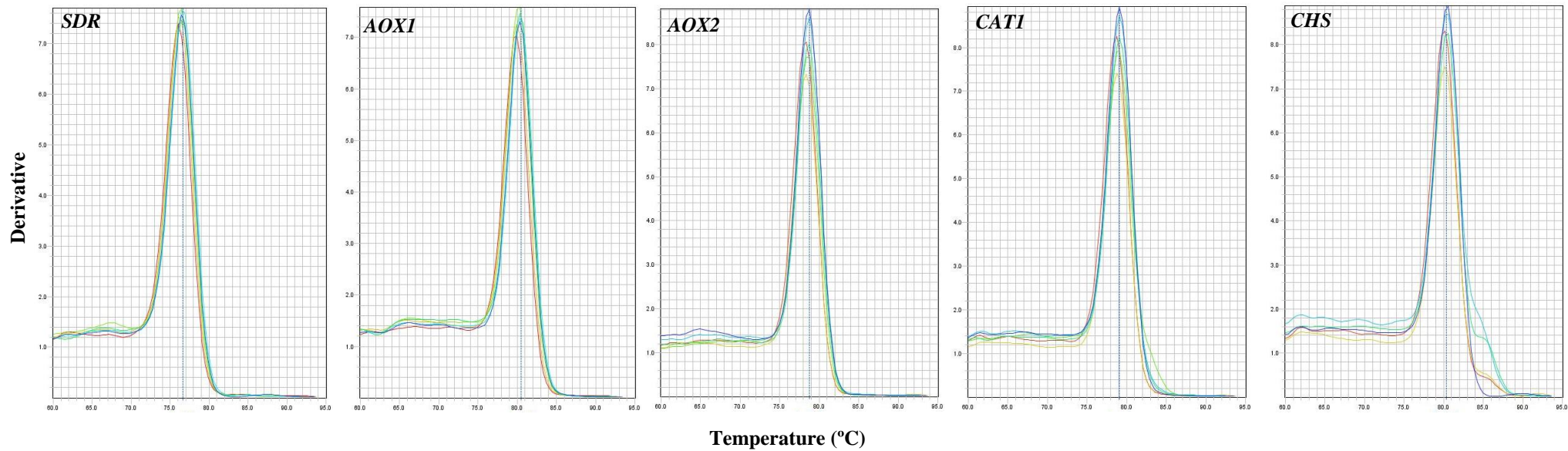

Supplement: S2 Figure — Melting curves of the 11 candidate reference genes and 4 target genes tested in heat assays. (PDF) [file pone.0115206.s002.pdf]
